# Commentary on “DeepSeek-R1 and GPT-4 are comparable in a complex diagnostic challenge: a historical control study”

**DOI:** 10.1097/JS9.0000000000003335

**Published:** 2025-09-08

**Authors:** Hanzhe Lv, Longhao Chen, Zhizhen Lv, Lijiang Lv

**Affiliations:** a The Third Affiliated Hospital of Zhejiang Chinese Medical University, Hangzhou, Zhejiang, China; b The Third School of Clinical Medicine (School of Rehabilitation Medicine), Zhejiang Chinese Medical University, Hangzhou, Zhejiang, China; c Research Institute of Tuina (Spinal disease), Zhejiang Chinese Medical University, Hangzhou, Zhejiang, China

*Dear Editor,*

We read with great interest the recent article titled “DeepSeek-R1 and GPT-4 are comparable in a complex diagnostic challenge: a historical control study” by Chan *et al*^[1]^, published in your esteemed journal. The authors compared the diagnostic performance of DeepSeek-R1, an open-source large language model (LLM), against GPT-4 using complex clinical cases. Their study demonstrated comparable diagnostic accuracy and highlighted DeepSeek-R1’s strengths in generating diverse differential diagnoses. We sincerely commend the authors for their valuable and insightful contribution. We also note that this commentary complies with the TITAN 2025 guidelines regarding the declaration and use of artificial intelligence in scientific manuscripts^[2]^.

Chan *et al* significantly advanced the literature by demonstrating that an open-source LLM can match a proprietary model (GPT-4) in diagnostic accuracy. They also noted DeepSeek-R1’s broader differential diagnosis capabilities, potentially enhancing clinical reasoning. These findings underscore the value of accessible AI tools and rigorous comparative evaluations. However, we suggest three additional considerations to enhance clinical applicability.

First, the issue of interpretability, which is essential for fostering clinician trust, was not explicitly addressed by Chan *et al*. Although DeepSeek-R1’s open-source nature suggests an inherent transparency, actual interpretability in clinical practice demands explicit evaluation. Brankovic *et al* recently proposed the clinician-informed XAI evaluation checklist with metrics (CLIX-M), aiming to systematically ensure that AI-driven clinical decision support systems are transparent and dependable^[3]^. Future studies could benefit from incorporating such frameworks and employing interpretability techniques (such as saliency mapping or decision-path tracing), thereby strengthening clinician trust and confidence in model recommendations.

Second, potential biases embedded within LLMs were not evaluated, an oversight that may unintentionally introduce clinical risks. Recent research by Yang *et al* underscored that even highly accurate AI diagnostic tools can harbor implicit biases, which can adversely affect performance in diverse populations and may widen disparities in healthcare outcomes^[4]^. Future evaluations would therefore benefit from rigorous assessments of model fairness – explicitly reporting subgroup-specific accuracy and applying targeted bias mitigation strategies – to ensure equitable care across all patient demographics.

Third, while historical datasets offer valuable insights, the model’s robustness in dynamic, real-world clinical environments remains unverified. Rosenthal *et al* recently advocated for dynamic and adaptive clinical trial designs for medical AI, noting that static datasets cannot fully reflect the complexities of practical medical scenarios^[5]^. Therefore, prospective evaluations of DeepSeek-R1 within actual clinical workflows are warranted. These evaluations should assess outcomes such as diagnostic timeliness, clinician acceptability, and the model’s resilience to real-time errors. Such real-world assessments would significantly enhance the model’s clinical relevance and usability.

Finally, three learning points emerge from this commentary. First, open-source LLMs such as DeepSeek-R1 can match proprietary models in complex diagnostic reasoning, thereby broadening access to AI-driven diagnostic support. Second, systematic interpretability audits – such as those proposed by the CLIX-M framework – are indispensable before clinical deployment. Third, fairness metrics and workflow-embedded prospective trials are critical for ensuring the safe, equitable, and robust implementation of AI models. Taken together, addressing interpretability, bias mitigation, and real-world validation will be pivotal for responsibly translating these promising AI tools from research settings into routine clinical practice. We deeply appreciate the foundational contributions by Chan *et al* and look forward to future advancements in this important field.

## Supplementary Material

## Data Availability

Not applicable.

## TITAN 2025 checklist

A completed TITAN 2025 checklist has been provided as a supplementary file (available at: http://links.lww.com/JS9/F50).
