# Supplementary material for: Commentary on “DeepSeek-R1 and GPT-4 are comparable in a complex diagnostic challenge: a historical control study”
Source: Int J Surg. 2025 Sep 8;112(1):1958–9. doi: 10.1097/JS9.0000000000003335 (PMC12825781; doi:10.1097/JS9.0000000000003335)
Supplement: Supplementary file 1 [file js9-112-1958-001.docx]

| **TITAN Guideline Checklist 2025** | | | |
| --- | --- | --- | --- |
| **Topic** | **Item** | **Description** | **Page number** |
| **Declaration of whether any AI was used in the research and manuscript development**  **State no, if that’s the case.**  **If yes, proceed to item 1a.** | 1 | Yes – AI was used for English-language polishing only. | 3 |
| **Purpose and Scope of AI Use** | 1a | AI was used for English language editing, grammar checking, and minor structural refinement during manuscript drafting and revision. Generative AI was employed exclusively for linguistic assistance during the writing phase. All content was critically reviewed by the authors to ensure accuracy and integrity. | 3 |
| **AI Tool(s) and Configuration** | 1b | OpenAI ChatGPT (GPT-4, July 2025 version) was used via the official cloud API. No plug-ins, fine-tuning, or third-party integrations were applied. Prompts were limited to paragraph-level refinements without automated content generation. | 3 |
| **Data Inputs and Safeguards** | 1c | Only anonymized manuscript text was provided to the AI. No patient data or protected health information was involved. All content complied with GDPR/HIPAA regulations, and no institutional approvals were required. | 3 |
| **Human Oversight and Verification** | 1d | The corresponding author and all co-authors reviewed each instance of AI-assisted output. Outputs were fact-checked and revised for academic tone and correctness. Any content with ambiguity or error was discarded. Limitations of AI tools were acknowledged throughout. | 3 |
| **Bias, Ethics and Regulatory Compliance** | 1e | AI use was limited to language editing, with no involvement in data analysis or clinical decision-making. No conflicts of interest or affiliations with AI vendors exist. Ethical use principles were followed throughout the manuscript preparation. | 3 |
| **Reproducibility and Transparency** | 1f | No AI-generated figures or datasets were created. All AI interactions were prompt-based and non-replicative. The prompts used were simple editorial requests (e.g., “Please improve grammar of this paragraph”). Full responsibility for the final manuscript rests with the authors. | 3 |
